# Prasiolin, a new UV-sunscreen compound in the terrestrial green macroalga *Prasiola calophylla* (Carmichael ex Greville) Kützing (Trebouxiophyceae, Chlorophyta)

**DOI:** 10.1007/s00425-015-2396-z

**Published:** 2015-09-10

**Authors:** Anja Hartmann, Andreas Holzinger, Markus Ganzera, Ulf Karsten

**Affiliations:** Institute of Pharmacy, Pharmacognosy, University of Innsbruck, Innrain 80-82/IV, 6020 Innsbruck, Austria; Institute of Botany, Functional Plant Biology, University of Innsbruck, Sternwartestrasse 15, 6020 Innsbruck, Austria; Institute of Biological Sciences, Applied Ecology and Phycology, University of Rostock, Albert-Einstein-Strasse 3, 18059 Rostock, Germany

**Keywords:** MAA (mycosporine-like amino acid) isolation, MAA purification, Structural elucidation, UV acclimation

## Abstract

**We introduced a novel combination of chromatographic techniques for the purification and analysis of a new UV-sunscreen mycosporine-like amino acid (MAA) in the terrestrial green alga*Prasiola calophylla***.

*Prasiola calophylla* (Carmichael ex Greville) Kützing (Trebouxiophyceae, Chlorophyta) is a typical member of terrestrial algal communities in temperate Europe, where it regularly experiences various stress conditions including strong diurnal and seasonal fluctuations in ultraviolet radiation (UVR). As a photoprotective mechanism *Prasiola* species and other related Trebouxiophycean taxa synthesize a mycosporine-like amino acid (MAA) as natural sunscreen whose chemical structure was unknown so far. In the present study a new methodological approach is described for the isolation, purification and structural elucidation of this novel sunscreen in *P. calophylla*. The new compound exhibits an absorption maximum at 324 nm (in the short ultraviolet-A), a molecular weight of 333 and a molecular extinction coefficient of 12.393 M^−1^ cm^−1^, and could be identified as *N*-[5,6 hydroxy-5(hydroxymethyl)-2-methoxy-3-oxo-1-cycohexen-1-yl] glutamic acid using one- and two-dimensional ^1^H and ^13^C-NMR spectroscopy. As trivial name for this novel MAA we suggest ‘prasiolin’. The ecologically essential function of prasiolin for UVR-protection in terrestrial algae of the Trebouxiophyceae is discussed.

## Introduction

The green algal genus *Prasiola* (Trebouxiophyceae, Chlorophyta) has a cosmopolitan biogeographic distribution from temperate to polar coastal and terrestrial environments in both the Northern and Southern Hemispheres (Rindi 2007). The ecology of its members is very diverse since few species grow under aquatic conditions in freshwater systems or in the supralittoral zone of rocky marine coasts, while most others represent a general and ubiquitous component of various terrestrial ecosystems (Friedmann 1969; Rindi and Guiry 2004; Rodriguez et al. 2007). Some *Prasiola* species, such as *P. crispa* ssp. *antarctica*, show even a symbiotic lifestyle with Ascomycota resulting in the lichen *Mastodia tessellata* (Perez-Ortega et al. 2010). Compared to aquatic environments, terrestrial *Prasiola* are exposed to harsher abiotic conditions, such as strong gradients in water potential between the terrestrial habitat (e.g. soil or rock surface) and the atmosphere, resulting in regular desiccation (Hunt and Denny 2008; Holzinger and Karsten 2013). In addition, terrestrial algae experience strong diurnal and seasonal fluctuations in insolation including UVR.

In many regions of the world UVR is enhanced due to anthropogenically caused stratospheric ozone loss, which is particularly strong during spring in Antarctica (‘ozone hole’) (e.g. Whitehead et al. 2000), and UVR additionally increases with altitude as documented for the Alps (Blumenthaler et al. 1996; Karsten 2008). The altitudinal effect is depending on the wavelengths, i.e. ultraviolet-B radiation (UV-B, 280–315 nm) is proportionally much stronger enhanced with increasing elevation than ultraviolet-A radiation (UV-A, 315–400 nm) (Blumenthaler et al. 1996). Both UV-A and UV-B represent a major stress factor for many phototrophic organisms in terrestrial ecosystems (Karsten 2008). Terrestrial algae face a photobiological dilemma, since solar radiation is essential for photosynthesis, and at the same time the UVR portion of the spectrum can negatively affect many physiological processes, mainly due to direct absorption by key biomolecules. UV-B, for example, is strongly absorbed by DNA/RNA and proteins causing conformational changes or even photo damage that can subsequently disturb vital metabolic functions such as transcription, DNA replication and translation (Buma et al. 1997). In the Antarctic terrestrial *P. crispa* ssp. *antarctica* DNA damage has been documented after exposure to a combination of natural and artificially enhanced UV-B (Lud et al. 2001).

However, if terrestrial algae are regularly confronted with UVR in their habitat they rely on a number of different physiological or biochemical mechanisms to mitigate or even avoid biologically harmful UVR effects to guarantee long-term survival (Karsten 2008). These include avoidance, numerous protective mechanisms and/or repair of essential biomolecules (for details see Karsten 2008 and references therein).

A key protective mechanism in many terrestrial algae is the biosynthesis and accumulation of UV-absorbing sunscreens, such as mycosporine-like amino acids (MAAs) as documented for various members of the Trebouxiophyceae (Karsten et al. 2005, 2007) and Streptophyta (Kitzing et al. 2014; Kitzing and Karsten 2015). MAAs are low-molecular-weight compounds with maximum absorption bands between 310 and 360 nm in the UV range (Cockell and Knowland 1999). Chemically MAAs represent a suite of closely related, colourless, water-soluble, polar and at cellular pH uncharged or zwitterionic amino acid derivatives that consist of aminocyclohexenone or aminocyclohexenimine rings (Karsten 2008 and references therein). So far, different MAAs have been identified in terrestrial cyanobacteria, green algae and fungi (Garcia-Pichel and Castenholz 1993; Gorbushina et al. 2003; Karsten et al. 2007).

During a study on the occurrence and function of MAAs in Antarctic macroalgae Hoyer et al. (2001) reported in *P. crispa* ssp. *antarctica* a high concentration of a single unique, but chemically unknown UV-absorbing substance with an absorption maximum at 324 nm. Gröniger and Häder (2002) confirmed the occurrence of this putative “324 nm-MAA” in the closely related *P. stipitata* from the supralittoral zone of the rocky island Helgoland (North Sea). Since species of the thalloid *Prasiola* are phylogenetically related to other terrestrial green algae with a vegetative coccoid or pseudo-filamentous morphology (Friedl and O’Kelly 2002), a screening on the occurrence of the 324 nm-MAA in these members of the Trebouxiophyceae was undertaken (Karsten et al. 2005). Using high pressure liquid chromatography (HPLC) the data again confirmed the presence of the same molecule in the tested Trebouxiophyceae, and preliminary UVR-exposure experiments indicated a strong accumulation of this compound (Karsten et al. 2005). Based on these results the effect of controlled UV-A and UV-B on photosynthetic performance, growth and the capability to synthesize this putative 324 nm-MAA was investigated in various other than in Karsten et al. (2005) screened terrestrial Trebouxiophycean green algae forming biofilms on building facades or growing on soil (Karsten et al. 2007). The identical UV-absorbing compound was assigned by HPLC in *Stichococcus* sp. and *Chlorella luteo*-*viridis* based on matching UV-spectra and retention times. Furthermore, UVR-exposure experiments resulted in its strong and dose-depending biosynthesis and accumulation, thus supporting the function as an UV sunscreen. More importantly, the increase in MAA concentration in *Stichococcus* sp. and *C. luteo*-*viridis* was reflected in a reduced UV-sensitivity of growth and photosynthesis, which well explains the conspicuous ecological success of many Trebouxiophycean green algae in the environmentally harsh terrestrial habitat.

Since the chemical structure of the putative 324 nm-MAA in *Prasiola* and related Trebouxiophycean genera is still not known, we developed a methodological approach to isolate, purify and elucidate the structure of this sunscreen compound. *Prasiola calophylla* was used as a model system because it is an abundant component of terrestrial algal communities in rather rainy and temperate regions of Europe (Rindi and Guiry 2004).

## Materials and methods

### Biological material

**Fig. 1:**
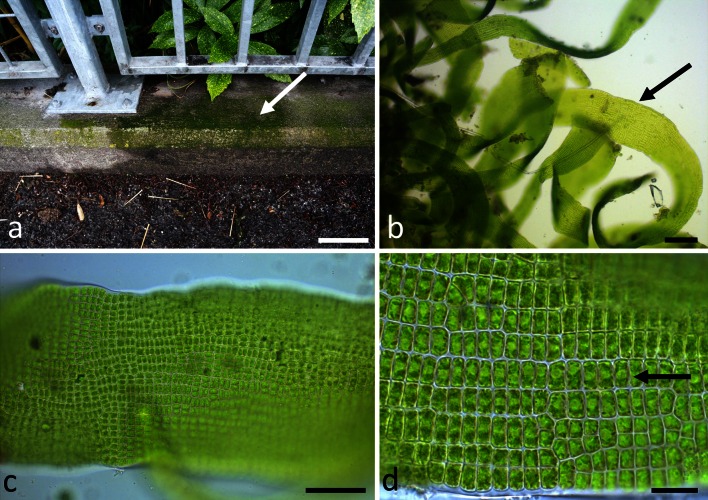
Habitat and morphology of the terrestrial *green* alga *Prasiola calophylla*. **a** Concrete wall with well-developed population of *Prasiola calophylla* (*arrow*); **b** overview of *ribbon-like curved* fronds (*arrow*); **c** individual thallus piece, ~160 µm broad; **d** details of thallus showing the cell morphology and arrangement of the cells in individual rows (*arrow*). *Bars* 10 cm (**a**), 100 μm (**b**), 50 μm (**c**) and 20 μm (**d**)

*Prasiola calophylla* (Carmichael ex Greville) Kützing (Trebouxiophyceae, Chlorophyta) was collected from a concrete basement of a metal fence at the Botanical Garden, University of Innsbruck (47°16′2″N, 11°23′34″O, 611 m above sea level) (Fig. 1a). This habitat was partially shaded. The thalli were wetted with tap water and scratched from the concrete surface using a spatula and pooled to about 10 g of wet weight. Fresh samples were investigated by a Zeiss Axiovert 200 M light microscope (Fig. 1b–d). The ribbon-like fronds were ~50–250 µm broad and curved, the individual cells of a thallus formed long rows (Fig. 1d). A sub-sample was send to Dr. Fabio Rindi, University of Ancona, Italy, for species identification (Rindi and Guiry 2004; Rindi et al. 2007).

### MAA extraction

Dried algal material was crushed to powder in a grinding mill prior to extraction using methanol/water (25:75, v/v) in an ultrasonic bath (35 kHz, Bandelin Sonorex, Berlin, Germany) at 45 °C for 2 h according the method of Tartarotti and Sommaruga (2002). After centrifugation at 3000 rpm (equal to 1000×*g*; Labofuge 400, Heraeus-Thermo, Braunschweig, Germany) for 10 min, the supernatant was collected and evaporated at 45 °C in a vacuum evaporator (Büchi, Flawil, Switzerland).

### MAA isolation

**Fig. 2:**
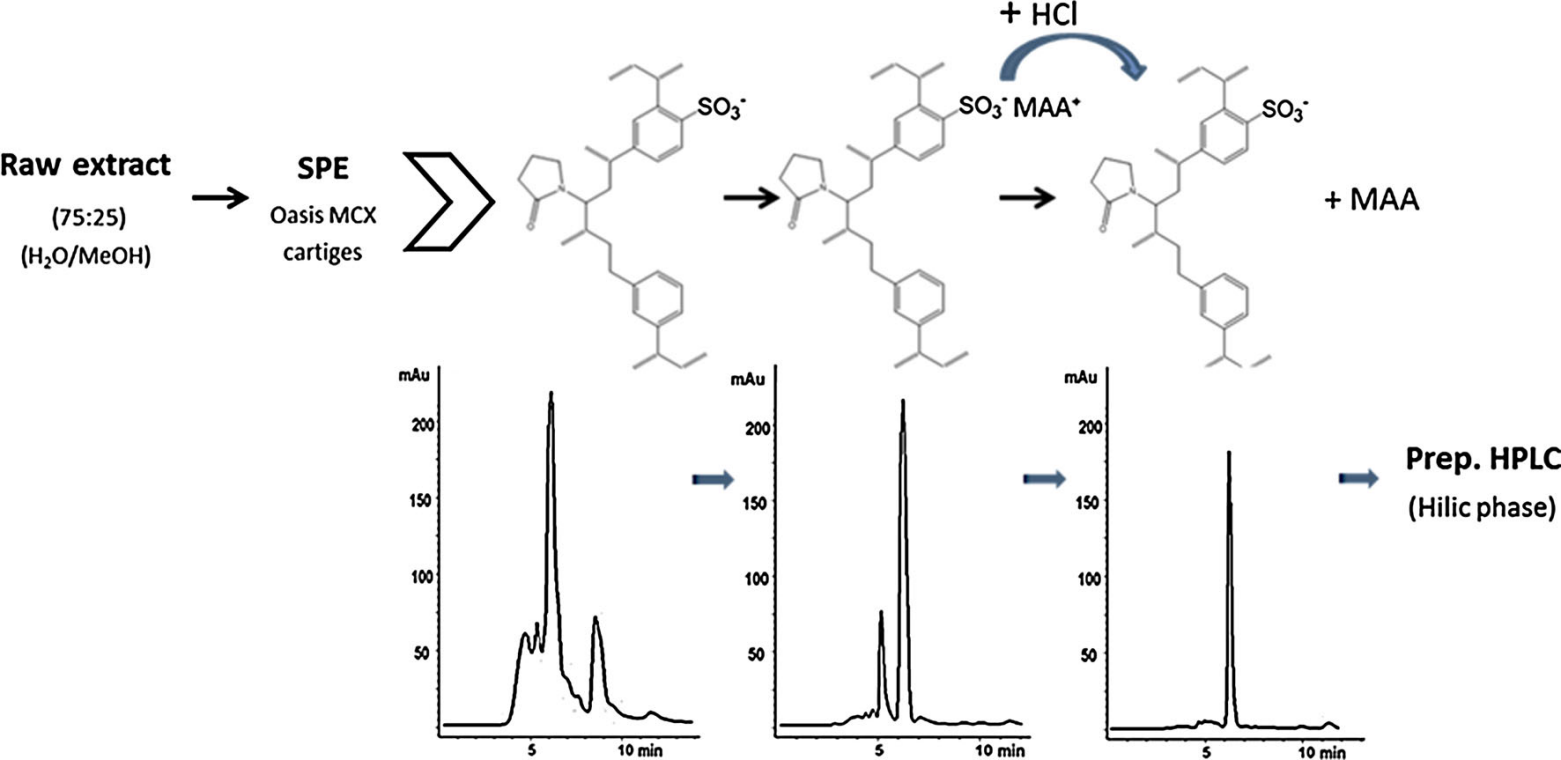
Schematic illustration of the isolation protocol for the novel MAA in the terrestrial green alga *Prasiola calophylla*

Dried extracts were dissolved in water and purified using Oasis MCX SPE cartridges (Waters Corporation, Milford, MA, USA) which represent a selective solid-phase extraction tool. The separation was based on a strong cation exchange resin, whereby the cartridges were first conditioned with methanol and water by rinsing with one column volume each, before applying the algal extract. After a washing step with water (two column volumes), MAA-rich fractions were eluted with 0.5 M HCl (two column volumes). The purification procedure was monitored by analysing solutions of each step by HPLC using a HILIC Poroshell 120 column (150 × 4.6 mm, 2.7 µm; Agilent, Waldbronn, Germany). Figure 2 demonstrates the purification on the SPE-cartridges including HPLC chromatograms. The dominant MAA was then isolated from the pre-purified extract by semi-preparative HPLC on an UltiMate 3000 preparative HPLC system (Dionex-Thermo Inc., Waltham, MA, USA). The optimum separation was carried out on a Luna 5 µ Hilic column 200Å (250 × 4.6 mm) from Phenomenex (Phenomenex, Aschaffenburg, Germany) by using a mobile phase consisting of (A) acetonitrile/water (9:1, v/v) with 5 mM ammonium acetate and (B) acetonitrile/water (1:1, v/v) with 5 mM ammonium acetate. A linear gradient was applied from 100 % mobile phase A to 30 % mobile phase A in 25 min, followed by a re-equilibration step of 15 min prior to the next injection. Detection was performed at 320 nm, the column was maintained at 30 °C and the flow rate was set to 1.0 mL min^−1^. The injected sample volume was 50 μL with a sample concentration of 20 mg mL^−1^. After approximately 10 injections 2.0 mg of a pure compound were obtained.

### LC–MS experiments for MAA mass determination

**Fig. 3:**
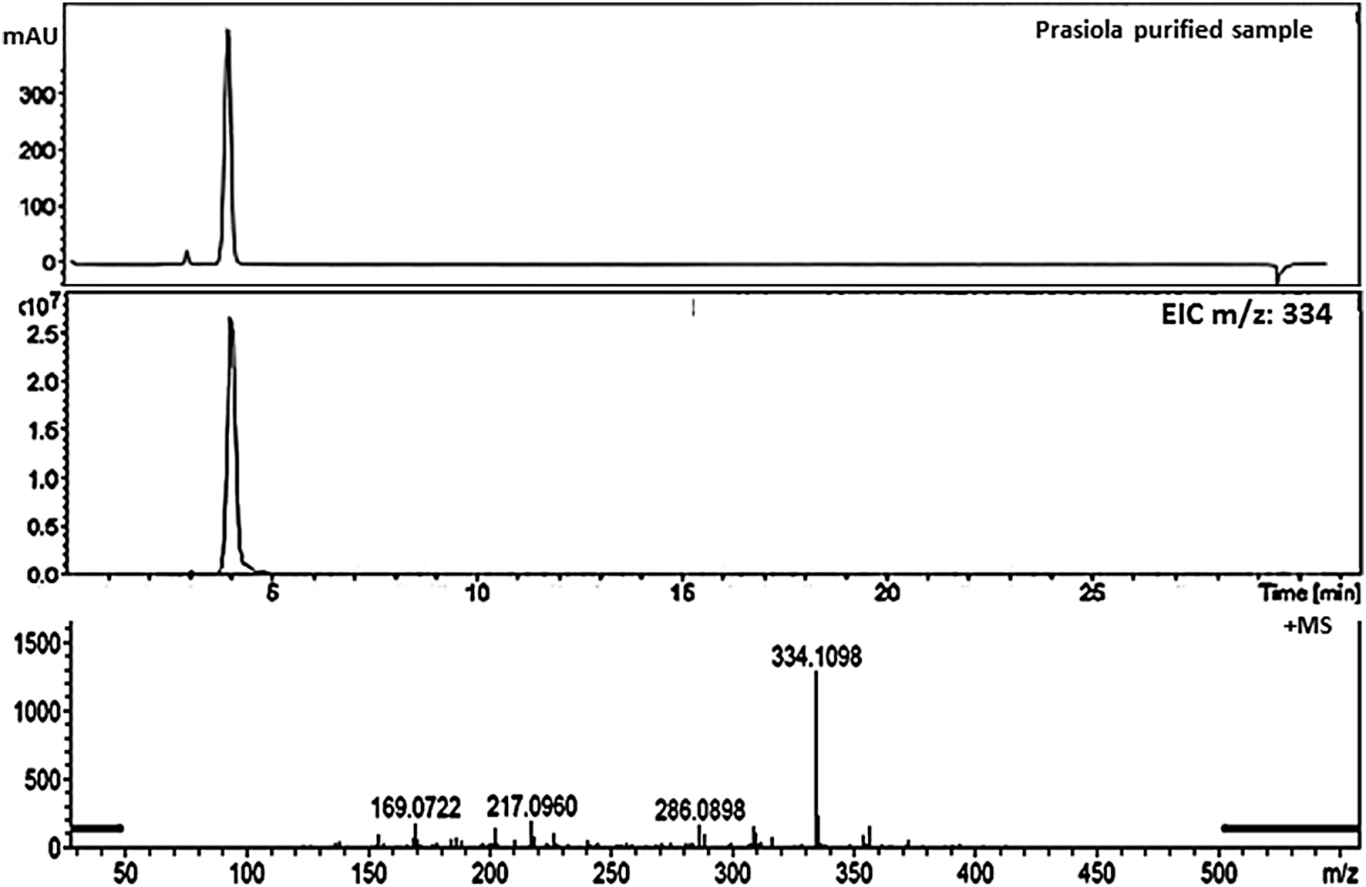
HPLC–MS data for molecular weight determination of the novel MAA in the terrestrial green alga *Prasiola calophylla*. *Top* HPLC chromatogram of the purified *Prasiola* extract. *Middle* and *below* Extracted ion chromatogram (*EIC*) and mass spectrum of the purified *MAA*, corresponding to an *m*/*z* value of [M+H]^+^, respectively

To determine the molecular weight of the isolated compound and to confirm peak purity HPLC–MS experiments were performed, using a 1100 HPLC system from Agilent (Agilent), coupled to an Esquire 3000 plus iontrap mass spectrometer (Bruker, Bremen, Germany). MS-Spectra were obtained applying alternating ESI mode and by setting the temperature to 350 °C, the nebulizer gas (nitrogen) to 40 psi, and a nebulizer flow (nitrogen) of 8 L min^−1^. Additionally, the exact mass of the compound was determined by analysing the sample on a micrOTOF-Q II MS (Bruker). Here the settings were: nebulizer gas, 5.8 psi (nitrogen); dry gas, 4.0 L min^−1^ (nitrogen); and dry temperature, 180 °C. Capillary voltage was 4.0 kV (positive ESI mode). The scanned mass range was between *m/z* 50 and 500 (Fig. 3).

### Structural elucidation of MAA

Nuclear magnetic resonance (NMR) spectra of the isolated compound were recorded at 25 °C on an Ultra-Shield 600 MHz instrument (Bruker) using the following experiments: ^1^H- and ^13^C-NMR, two-dimensional correlation spectroscopy (2D COSY), heteronuclear multiple quantum coherence (HMQC) and heteronuclear multiple bond coherence (HMBC). All samples were dissolved in deuterated water (D_2_O) containing tetramethylsilane (TMS) as internal standard (Euriso-Top, Saint-Aubin Cedex, France). ^1^H and ^13^C-NMR data of the isolated compound are summarized in Table 1.

**Table 1:** ^1^H and ^13^C NMR chemical shifts (in ppm) and proton coupling constants (Hz, in parentheses) of the novel MAA *N*-[5,6 hydroxy-5(hydroxymethyl)-2-methoxy-3-oxo-1-cycohexen-1-yl] glutamic acid (prasiolin) from the terrestrial green alga *Prasiola calophylla*

Prasiolin		
	^**13**^ **C**	^**1**^ **H**
1	192.10	–
2	133.05	–
3	159.55	–
4	37.94	2.80 (d, 17.7 Hz), 3.01 (d, 17.5 Hz)
5	75.85	–
6	74.91	4.29 (s)
7	68.03	3.61 (d, 11.6 Hz), 3.69 (d, 11.6 Hz)
8	63.33	3.48 (s)
9	57.51	3.76 (dd, 4.8/7.2 Hz)
10	177.83	–
11	29.69	2.06 (dt, 7.2, 14.9), 2.14 (dtd, 4.9, 7.6,12.5)
12	36.16	2.38 (m)
13	183.69	–

Spectra were recorded in D_2_O at 600 MHz. The molecular weight is 333 g mol^−1^

### HPTLC analysis of the MAA

High performance thin layer chromatography (HPTLC) experiments were performed to confirm our NMR results. Stock solutions of the crude extract (2 mg mL^−1^), glutamic acid (1 mg mL^−1^), and the purified sample (0.5 and 1 mg mL^−1^) were prepared. 30 µL of the crude extract, 10 µL of the glutamic acid solution and 20 µL of the purified sample were applied on a HPTLC plate (Merck, Darmstadt, Germany) using a Linomat V applicator (Camag, Muttenz, Switzerland). The bands were spotted with 10 mm width, spaced 10 mm from each other and 10 mm apart from the bottom edge of the plate. The plate was developed using the Automatic Developing Chamber ADC 2 (Camag) previously saturated with butanol:water:acetic acid (6:2:2, by vol.). Respective bands became visible by spraying the plate with 1 % ninhydrin dissolved in ethanol (cf. Fig. 5).

### Determination of the molar extinction coefficient

One milligramme of the isolated compound was dissolved in 10 mL distilled water, and this solution further diluted until the extinction at 324 nm was below 1.0; respective experiments were conducted on a UV-1800 photometer (Shimadzu, Kyoto, Japan). NMR experiments revealed that the isolated MAA contained free glutamic acid as an impurity. The respective ratio was determined by qNMR based to the integrals of known signals of the MAA and impurity in relation to the internal standard tetramethylsilane (TMS). This is a well-established approach to assess the purity of a substance (Simmler et al. 2014). The actual content of MAA in the sample was then considered for calculating the molar extinction coefficient.

## Results

The crude extract of *Prasiola calophylla* was investigated via HPLC–MS and revealed a dominant peak representing a substance with an absorption maximum at 324 nm and a molecular weight of 333 (Fig. 3). The chromatographic behaviour and UV-spectra provided strong indication for the presence of an MAA (Hoyer et al. 2001; Karsten et al. 2005).

Earlier attempts on purifying this compound by reversed phase HPLC were not successful, although receiving fractions showing a single peak only. Amino acids as well as sugars were co-eluting (data not shown), and this made a further chemical characterization of the target substance impossible. Since the molecular weight of the new substance did not match with any previously published MAA data, chemical structure elucidation of this compound by NMR was required. Therefore, as next step the isolation and purification of the putative MAA became necessary, which was conducted by combining pre-purification on selective solid-phase extraction (SPE) cartridges with preparative HPLC carried out on a hydrophilic interaction liquid chromatography (HILIC) column (Fig. 2). The latter is particularly designed to effectively separate small polar compounds. This new methodological approach resulted in a purified sample, whose structural elucidation was finally possible.

**Fig. 4:**
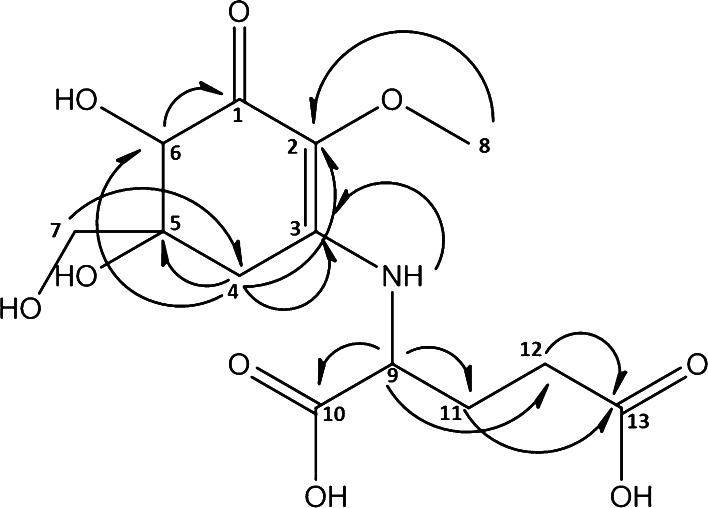
Chemical structure of the novel MAA *N*-[5,6 hydroxy-5(hydroxymethyl)-2-methoxy-3-oxo-1-cycohexen-1-yl] glutamic acid (prasiolin) from the terrestrial green alga *Prasiola calophylla*. Long-range correlations (*arrows*) were deduced from 2D-NMR experiments

The purified compound was analysed using ^1^H and ^13^C-NMR spectroscopy. By means of one- and two-dimensional NMR its structure was confirmed to be a novel MAA, namely *N*-[5,6 hydroxy-5(hydroxymethyl)-2 methoxy-3-oxo-1 cycohexen-1 yl] glutamic acid (Fig. 4). The respective ^1^H and ^13^C NMR data are given in Table 1. The individual ^1^H and ^13^C NMR signals were assigned according to ^1^H,^1^H COSY, and ^1^H,^13^C correlation experiments (HMQC, HMBC). In analogy to other well-characterized MAAs, where the biological source organism provided the trivial name of the respective compound, we suggest ‘prasiolin’ to name the new UV-absorbing substance in *P. calophylla*.

The isolated fraction was found to contain glutamic acid as well. In order to determine the molar extinction coefficient of the new MAA, the ratio of MAA to glutamic acid was determined by qNMR. It was found to be 10.0:1.9 (glutamic acid:MAA). Accordingly, the MAA concentration was corrected by this factor and the molar extinction coefficient resulted in a value of 12.393 M^−1^ cm^−1^.

**Fig. 5:**
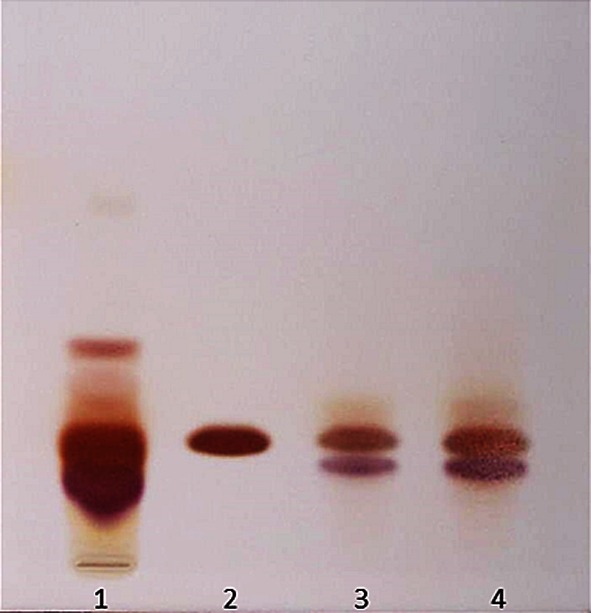
HPTLC separations of *Prasiola calophylla* crude extract (*lane 1*), glutamic acid (*lane 2*), purified MAA prasiolin in two concentrations (0.5 mg mL^−1^, *lane 3*; 1 mg mL^−1^, *lane 4*); mobile phase: BuOH:H_2_O:acetic acid (6:2:2, by vol.); reagent, 1 % ninhydrin dissolved in ethanol; stationary phase, silica gel F254

To verify of our purification strategy (Fig. 2) as well as HPLC–MS (Fig. 3) and NMR (Fig. 4) data, we additionally performed an HPTLC experiment to visualize the content of glutamic acid in the isolated sample. The crude *Prasiola* extract, glutamic acid and the isolated sample (in 2 concentrations) were applied on the TLC plate (Fig. 5). After spraying with ninhydrin dye glutamic acid could be confirmed as impurity due to matching Rf values. While co-eluting compounds such as amino acids or sugars could not be detected by HPLC using a diode array detector (DAD), the simple separation on silica-based HPTLC plates in combination with a suitable spray reagent enabled a clear differentiation of glutamic acid and the novel MAA.

## Discussion

Taking into account that the analysis of MAAs is almost exclusively carried out using reversed-phased HPLC with DAD detection (Karsten et al. 2009 and references therein), our study should draw attention to possible pitfalls in previously described MAA characterization and isolation protocols. The extremely strong absorption of these compounds (especially in the specific range from 310 to 360 nm) might pretend pure compounds, but other substances (e.g. amino acids, sugars) are either not detected under the given circumstances (type of detector, selected wavelength) or co-elute. In the current study, we utilized a novel combination of techniques (SPE and preparative HPLC on a HILIC phase) for the purification of an MAA occurring in *P. calophylla*. But even then the apparently (and chromatographically) pure compound contained a large proportion of a second substance (glutamic acid). Thus, for meaningful conclusions regarding the underlying chemical structure NMR studies are inevitable. However, already a simple and fast preliminary test by (HP)TLC could reveal possible impurities. Our efforts finally resulted in the isolation and elucidation of a novel MAA, *N*-[5,6 hydroxy-5(hydroxymethyl)-2-methoxy-3-oxo-1-cycohexen-1-yl] glutamic acid, which was named prasiolin.

The high content of glutamic acid in the purified sample might have several reasons (see below), but the most obvious one is a possible degradation of the molecule. Re-recorded ^1^H NMR spectra of the sample solution (in deuterated water) showed no changes within several hours and additional HPLC–MS experiments did not reveal degradation products like gadusol, the core structure of this MAA (Bandaranayake 1998). However, it is possible that MAAs with an oxo-carbonyl structure are generally less stable then the much better studied amino-cyclohexenimine structures. This could be an explanation why only a few oxo-carbonyl MAAs are known till date. Further investigations in this direction, e.g. by conducting stability studies are definitely required.

MAAs are chemically related to fungal mycosporines, which were first described from sporulating mycelia (Leach 1965; Favre-Bonvin et al. 1976). The various MAA structures result from N-substitutions of different amino acid moieties to the cyclohexenone and cyclohexenimine chromophore, respectively. At present, there are only 2 known aminocyclohexenone-derived MAAs such as mycosporine-glycine and mycosporine-taurine, which typically exhibit their absorption maximum in the UV-B range (Carreto and Carignan 2011). Both compounds can be considered to be Schiff bases (enamino ketones) as they possess a cyclohexenone ring system linked with an amino acid (oxocarbonyl-MAAs) (Carreto and Carignan 2011). The novel MAA ‘prasiolin’ from *P. calophylla* is chemically closely related to mycosporine-glycine and mycosporine-taurine, and hence represents an example for a rather rare MAA structure in a terrestrial alga. All the other described MAAs are derivatives of the aminocyclohexenimine structure (Carreto and Carignan 2011).

MAAs are regarded to be the strongest UVR-absorbing compounds in nature (Karsten 2008; Carreto and Carignan 2011). They are proposed to function as passive shielding solutes by dissipating the absorbed short wavelength radiation energy in the harmless form of heat without generating photochemical reactions (Bandaranayake 1998). These biomolecules exhibit extremely high absorptivity for UV-A and UV-B (molar extinction coefficients between 28,000 and 50,000) (Carreto and Carignan 2011), and although the measured molar extinction coefficient of 12.393 M^−1^ cm^−1^ for the novel MAA ‘prasiolin’ is lower than those of the known compounds, it is in the same range of magnitude. There are various reports that MAAs exhibit a high degree of photostability, which is a prerequisite for their sunscreen function (Conde et al. 2000).

The UV-screening function of MAAs has been inferred in numerous red macroalgae from a decrease in concentration with increasing depth (Hoyer et al. 2001). Supra- and eulittoral red algal species such as members of the genus *Porphyra* typically experience the strongest UVR, and consequently synthesize and accumulate high MAA contents, which generally are positively correlated with the natural UV doses (Huovinen et al. 2004). In contrast, other red algal taxa growing in the deep waters are biochemically not capable of producing MAAs (Hoyer et al. 2001; Karsten 2008). In this context, *Prasiola* species from Antarctica, the Arctic and Helgoland (North Sea, Germany) have also been described as one of the few green macroalgal genera exhibiting always enhanced MAA contents (Hoyer et al. 2001; Gröniger and Häder 2002; Karsten et al. 2009). In addition, Gröniger and Häder (2002) investigated the wavelength-dependent induction of the MAA biosynthesis in *P. crispa* using simulated UVR in combination with an array of cut-off filters, demonstrating wavelengths between 320 and 335 nm to be particularly effective. The screening function of MAAs was experimentally evaluated for various cyanobacteria (Garcia-Pichel and Castenholz 1993), and these authors documented that supplemental UVR led to a strong induction in MAA production resulting in attenuation of UVR effects.

Besides the role as natural UV-sunscreen compounds, some MAAs such as mycosporine-glycine exhibit also a moderate antioxidant activity (Dunlap and Yamamoto 1995). In addition, the biochemical precursor of MAAs, 4-deoxygadusol shows strong antioxidant activity (Dunlap et al. 1998). Both mycosporine-glycine and 4-deoxygadusol possess the cyclohexenone ring system, and hence it is possible that the novel “prasiolin” also has such an activity.

The ephemeral, tufty *Prasiola* species are ecologically interesting because of their capability to grow outside the aquatic milieu on bark, soil and rock, as well as in the supralittoral zone of marine rocky shores. In Antarctica and the Arctic, members of this genus always prefer habitats rich in nitrogen containing faeces of birds such as penguin colonies or underneath or near seagulls (Holzinger et al. 2006). In the presently investigated *P. calophylla*, nitrogen input, for example, by dog excrements is likely one factor supporting the abundant growth. Considering a relation between the MAA contents and nitrogen availability in different species of the red alga *Porphyra* (Korbee et al. 2005), as well as a nitrogen-dependency of photoacclimation in *Ulva rotundata* (Henley et al. 1991), it becomes obvious that this nutrient might be a critical factor for the photophysiological performance of *Prasiola* under terrestrial conditions since nitrogen is an essential element of the novel MAA ‘prasiolin’. When living under terrestrial conditions *Prasiola* species have to cope with strong amplitudes of the prevailing abiotic parameters. Seasonal studies on an Antarctic *Prasiola* species indicated some variation in the MAA concentrations, going along with high minimum steady-state amounts (Jackson and Seppelt 1997). In addition to the capability to synthesize MAAs, members of this genus have developed various morphological, physiological and biochemical protective mechanisms such as thick cell walls as mechanical barriers (Jacob et al. 1992), rather insensitive organelles under UVR (Holzinger et al. 2006) and the formation of polyols such as sorbitol to compensate water potential differences (Jacob et al. 1991).

In conclusion, in the present study a new methodological approach for the isolation and purification of a new UV-sunscreen compound in the terrestrial *P. calophylla* was successfully applied. This strategy opens new possibilities for future investigations on uncommon MAAs in sun-exposed and UVR-tolerant organisms.

### *Author contributionsstatement*

Anja Hartmann: undertook all practical experiments, processed the data, prepared most figures and table, edited the manuscript; Andreas Holzinger: collected the material, prepared Fig. 1, edited the manuscript; Markus Ganzera: supervised Anja Hartmann, helped with all methodological approaches and interpretation of the data, did final editing of the manuscript; Ulf Karsten: developed the scientific question, prepared first draft of manuscript.

## Abbreviations

HPTLC: High performance thin layer chromatography
MAA: Mycosporine-like amino acid
UVR: Ultraviolet radiation
